# Identification and validation of serum autoantibodies in children with B-cell acute lymphoblastic leukemia by serological proteome analysis

**DOI:** 10.1186/s12953-021-00184-w

**Published:** 2022-02-02

**Authors:** Runhong Yu, Shiwei Yang, Yufeng Liu, Zunmin Zhu

**Affiliations:** 1 Institute of Hematology, Henan Provincial People’s Hospital, 7 Weiwu Road, Jinshui District, Zhengzhou, Henan, 450003 China; 2 Henan Key laboratory of Stem Cell Differentiation and Modification, Henan Provincial People’s Hospital, Zhengzhou, Henan China; 3 Department of Pediatrics, The First Affiliated Hospital of Zhengzhou University, 1 Jianshe Road, Erqi District, Zhengzhou, Henan, 450052 China; 4 Department of Hematology, People’s Hospital of Zhengzhou University, Henan Zhengzhou, China

**Keywords:** B-cell ALL, Autoantibody, Acute lymphoblastic leukemia, Serological proteome analysis, Children

## Abstract

**Background:**

B-cell acute lymphoblastic leukemia (B-ALL) is the most common malignancy of childhood. Even though significant progresses have been made in the treatment of B-ALL, some pediatric B-ALL have still poor prognosis. The identification of tumor autoantibodies may have utility in early cancer diagnosis and immunotherapy. In this study, we used serological proteome analysis (SERPA) to screen serum autoantibodies of pediatric B-ALL, aiming to contribute to the early detection of B-ALL in children.

**Methods:**

The total proteins from three pooled B-ALL cell lines (NALM-6, REH and BALL-1 cells) were separated using two-dimensional gel electrophoresis (2-DE), which was followed by Western blot by mixed serum samples from children with B-ALL (n=20) or healthy controls (n=20). We analyzed the images of 2-D gel and Western blot by PDQuest software, and then identified the spots of immune responses in B-ALL samples compared with those in control samples. The proteins from spots were identified using mass spectrometry (MS). The autoantibodies against alpha-enolase (α-enolase) and voltage-dependent anion-selective channel protein 1 (VDAC1) were further validated in sera from another 30 children with B-ALL and 25 normal individuals by the use of enzyme-linked immunosorbent assay (ELISA). The protein expression levels of the candidate antigens α-enolase and VDAC1 in B-ALL were thoroughly studied by immunohistochemical analysis.

**Results:**

Utilizing the SERPA approach, α-enolase and VDAC1 were identified as candidate autoantigens in children with B-ALL. The frequencies of autoantibodies against α-enolase and VDAC1 in children with B-ALL were 27% and 23% by using ELISA analysis, respectively, which were significantly higher than those in normal controls (4% and 0, *p*<0.05). Immunohistochemical analysis showed the expression of α-enolase and VDAC1 was positive in 95% and 85% of B-ALL patients, respectively, but negative expression levels were showed in the control group.

**Conclusions:**

This study incidated that α-enolase and VDAC1 may be the autoantigens associated with B-ALL. Therefore, α-enolase and VDAC1 autoantibodies may be the potential serological markers for children with B-ALL.

**Supplementary Information:**

The online version contains supplementary material available at 10.1186/s12953-021-00184-w.

## Background

Acute lymphoblastic leukemia (ALL) is the most common type of neoplasms in childhood (children aged 0–14 years), and its incidence peaks between the ages of 1 and 4 years [1, 2]. 85% of pediatric ALL patients are of the B-cell lineage, and 15% are of the T-cell lineage [3]. Currently, bone marrow aspiration is used for diagnosis. However, because of the substantial damage this approach causes, the majority of children exhibit significant psychological pressure regarding this examination. In recent years, with the administration of stronger chemotherapy, the prognosis of ALL has been greatly improved, while approximately 20% of patients with ALL still experience relapse, despite intensive chemotherapy; additionally, traditional chemotherapy drugs cause toxic side effects, which may lead to death [1, 3]. Therefore, there is an urgent need to find biomarkers which are noninvasive and specific for diagnosis and targeted therapy of pediatric B-ALL.

Mutated or aberrantly expressed proteins are produced in the process of tumorigenesis and progression of neoplasms, and these proteins are capable of eliciting an immunological reaction, which leads to the generation of autoantibodies [4]. At present, vailable technologies couldn’t detect tumor-associated antigens (TAAs) at low levels during the early stages of tumor growth; nevertheless, a large amount of autoantibodies which are existent may be detected for months or years before the clinical confirmation of premalignant cancer [5]. Autoantibodies have been suggested in the serum of patients with a wide variety of tumor and have shown possible for use as biomarkers for tumor diagnosis [6]. A large number of studies have confirmed that autoantibodies were also involved in the development of tumors [7–10]. For example, serum p53 antibody was detected in the serum of about 30% patients with colon cancer, lung cancer, breast cancer and hepatocarcinoma [11]. The diagnostic specificity of serum p53 antibody may be as high as 96% [11].

Therefore, we concentrated on screening autoantibodies as serum biomarkers of B-ALL using serological proteome analysis (SERPA) with combination of two-dimensional gel electrophoresis (2-DE), immunoblotting and MS in this study. In recent decades, autoantibodies against TAAs were identified by SERPA in various diseases, containing hepatocellular carcinoma [12], colorectal cancer [10], cholangiocarcinomas [13], lung cancer [14], gallbladder carcinoma [15], prostate cancer [16], type 1 diabetes [17] and primary open angle glaucoma [18]. However, as far as we know, there is no report on the screening of autoantibodies in children with B-ALL. The examination of serum autoantibodies could not only contribute to the diagnosis of B-ALL, but also faciliate the development of targeted therapy. The main objective of this study was to screen new TAAs in B-ALL cell lines and confirm related autoantibodies in the serum from children with B-ALL by applying SERPA.

## Materials and methods

### Participants

The participants were recruited from the First Affiliated Hospital of Zhengzhou University. The study was performed according to the Declaration of Helsinki and was approved by the Institutional Ethics Committee of the Department of Medicine of the First Affiliated Hospital of Zhengzhou University. Informed consent in writing was obtained from the parents or guardians before the initiation of this study. The primary ALL diagnosis was based on 2016 WHO classification. Age-matched healthy controls were recruited from hospital outpatient clinics. All participants were younger than 14 years of age.

Four groups in two independent serum sample stages (discovery and validation) were used for this study (Table 1). Sera and bone marrow (BM) were obtained from 20 children with B-ALL at the time of diagnosis. Sera from 20 healthy children were used as control. In addition to the 40 specimens for SERPA, 55 independent serum specimens including 30 B-ALL and 25 healthy individuals were collected for ELISA validation. Basic clinical characteristics of patients and control subjects were described in Table 1. Moreover, bone marrow smears of 10 fully recovered patients with acute immune thrombocytopenia were collected for immunohistochemical analysis. All serum samples were stored at -80℃ until analysis was performed. All serum and BM samples were discarded after clinical use.

**Table 1:** Basic clinical characteristics of patients and control subjects

	Discovery stage		Validation stage	
	B-ALL	Healthy children	B-ALL	Healthy children
Number	20	20	30	25
Male	10	10	18	13
Female	10	10	12	12
Median age, years (range)	4(2 months-13 years)	6(1year-14 years)	4(2 months-13 years)	6(1year-14 years)

### Cell culture and cell extracts

Three human B-ALL cell lines, namely, NALM-6, REH and BALL-1 cells, were obtained from DSMZ (Deutsche Sammlung von Mikroorganismen und Zellkulturen, Germany), the Cell Bank of the Chinese Academy of Sciences (Shanghai, China) and the Institutes of Biomedical Sciences of Fudan University, respectively. The B-ALL cell lines were cultured in RPMI-1640 medium supplemented with 10% fetal bovine serum (BSA, Gibco; Thermo Fisher Scientific, Inc.), 100 U/ml streptomycin and 100 U/ml penicillin. All the cells were incubated at 37 °C in an atmosphere of 5% CO_2_. The total proteins were extracted from the cell lines. The cells were harvested and washed 3 times with phosphate-buffered saline (PBS) at 800 × rpm for 10 min and incubated for 30 min at 4 °C in SF cell lysis buffer (7 M urea, 40 mM Tris, 2 M thiourea, 2% CHAPS and 1% protease inhibitor cocktail (Roche, Germany)), followed by sonication at 20% amplitude (3 × 10 s). The cell lysates were then spun at 14,000 × rpm for 40 min, and the supernatants were collected. The proteins from the cell lines were purified by the 2-D Clean up kit (GE Healthcare). The final protein concentration was quantified by Bradford assay. Proteins were extracted from all three B-ALL cell lines as described above. Finally, equal amounts of proteins were mixed for further experiments.

### Two-dimensional gel electrophoresis (2-DE) and Western blotting

Protein extracts (200 µg) from cultured cells were lysed in rehydration buffer (2 M thiourea, 7 M urea, 2% CHAPS, 0.5% (v/v) immobilized pH gradient (IPG) buffer at pH 3-10, 1.5% (w/v) DTT and 0.002% bromophenol blue), which was used for passive rehydration of 7-cm, pH 3–10 nonlinear IPG strips (Bio-Rad, USA) by incubation at room temperature for 12 h in a strip holder; then, the samples were subjected to isoelectric focusing gel electrophoresis (the first-dimension gel). IEF was conducted at 50 µA/IPG strip, 250 V for 1.5 h, 1,000 V for 1 h, 4,000 V for 2 h, and 4000 V for 32,000 V-h. After focusing, the IPG strips were incubated in equilibration buffer (50 mM Tris-HCl buffer (pH 8.8), 6 M urea, 30% glycerol, 2% sodium dodecyl sulfate (SDS) and 0.002% bromophenol blue) with 1% DTT for 15 min and then in equilibration buffer with 2.5% iodoacetamide for 15 min (Bio-Rad protocol). The treated gel strips were loaded onto a second-dimensional gel and subjected to 10 mA/gel. At this point, the gels were stained using Coomassie blue dye method, by which proteins were visualized, or the proteins were transferred onto polyvinylidene fluoride (PVDF) membranes. After transfer, the PVDF membranes were blocked with blocking buffer (5.0% nonfat milk in Tris-buffered saline containing 0.1% Tween-20; TBST) and then incubated overnight at 4 °C with diluted sera from B-ALL patients (n=20) or normal controls (n=20) as primary antibodies at a 1:200 dilution. After washing, the membranes were incubated with horseradish peroxidase (HRP)-conjugated goat anti-human IgG (GE, USA) as a secondary antibody at a 1:10,000 dilution for 1.0 h at room temperature. The immunoreactive spots on the PVDF membranes were detected by an enhanced chemiluminescence kit (ECL Plus™ Western Blotting Detection kit, GE Healthcare, USA) according to the manufacturer’s instructions. The images were acquired using the Luminescent Image Analyzer LAS-3000 v2.2.

### In-gel digestion

The images of 2-D immunoblot were compared with the Coomassie stained images, by which the corresponding immunoreactive protein spots on the 2-D gel were identified with an ImageMaster 2D Elite 4.01 Software. The protein spots reacting with sera from B-ALL patients, but not with control sera, were excised manually from the Coomassie-stained gels. The excised gel fragments were destained with 200 µl of destainer, followed by the addition of 200 µl of 100% acetonitrile. After acetonitrile was cleared, the dried gel slices were digested with 0.01 µg/µl trypsin in 20 mM NH_4_HCO_3_ overnight at 37 °C. Peptides were extracted with extraction buffer (5% formic acid in 50% acetonitrile) and incubated for 30 min. This procedure was repeated twice, and the extracted peptides were pooled and concentrated to complete dryness before Mass spectrometry (MS) analysis.

### MS analysis

Each peptide sample was covered with 0.8 µL of 5 mg/ml CHCA (α-cyano-4-hydroxycinnamic acid) solubilized in 50% acetonitrile and 0.1% trifluoroacetic acid and then spotted on a MALDI plate. The spotted samples were submitted for data acquisition on a 5800 MALDI-TOF/TOF mass spectrometer (AB SCIEX, CA). MS spectra were acquired from 700 to 3600 m/z for a total of 1000 laser shots. Laser intensity remained fixed for all the analyses. MS/MS analyses were performed using 2 kV collision energy with air as CID gas. Metastable ions were suppressed for a total of 1000 laser shots. Spectra analysis was performed manually.

### Data analysis

All the MS/MS spectra were searched against the NCBI database using the Mascot server (version 2.3.2.0, Matrix Science, London, UK) search engine with scores of proteins above 71. The search parameters for the searches were as follows: NCBI database (created in October 2021) restricted to *Homo sapiens*; enzyme: trypsin; max missed cleavages: 1; peptide tolerance: 100 ppm; MS/MS tolerance: 0.6 Da; variable modifications: oxidation (M). All the MS/MS spectra were manually verified.

### ELISA for autoantibodies

The antigenic proteins α-enolase and VDAC1 were diluted to final of 1.0 µg/ml. α-enolase and VDAC1 were incubated in 96-well microplates in coating buffer at 4 °C overnight, then free unbound sites were blocked with 1% BSA for 2 h at 37 °C. The plates were incubated with human serum samples diluted at 1:100 with PBS for 1 h at 37 °C, then they were washed and incubated with 100 µl of horseradish peroxidase (HRP)-conjugated goat anti-human IgG diluted at 1:5000 with PBS (Santa Cruz Biotechnology) for 30 min at 37 °C. TMB substrate was added and incubated for 5 min at room temperature, and this reaction was terminated by adding the stop solution. The absorbance was measured by spectrophotometry at a wavelength of 450 nm. The cutoff value designating positive reaction was the mean optical density (OD) of 25 normal children sera plus 3 standard deviations (SD).

### Immunohistochemical analysis of α-enolase and VDAC1

We assessed the protein level of α-enolase and VDAC1 in 20 newly diagnosed B-ALL and 10 fully recovered acute immune thrombocytopenia children by immunohistochemical analysis. We harvested BM of children with acute immune thrombocytopenia more than 2 weeks after dexamethasone withdrawal. A few drops of BM were smeared directly onto slides, and BM slides were fixed with 4% paraformaldehyde fixative solution. The blocked sections were incubated with primary antibodies against α-enolase (Santa Cruz, dilution 1: 200) and VDAC1 (Abcam, dilution 1: 200) overnight at 4 °C, and then they were incubated with secondary antibodies using polyperoxidase-conjugated anti-mouse/rabbit IgG (GE Healthcare, dilution 1:200) at room temperature for 30 min. The BM slides were incubated with freshly prepared 3,3′-diaminobenzidine working solution at room temperature for 5 min, counterstained with hematoxylin, and dehydrated. The distribution of leukemia cell staining was scored as 0 (< 10% cell staining), 1 (10~40% cell staining), 2 (40~70% cell staining) or 3 (≥ 70% cell staining). The staining intensity of leukemia cells scored as 0, 1, 2 and 3 indicated negative, light yellow, brownish yellow and brown staining in the cytoplasm and/or nucleus, respectively. Total scores of 0-1, 2, 3-4, and 5-6 indicated (-), (+), (+ +) and (+ + +), respectively. In this study, (+-+++) was classified as positive expression of α-enolase and VDAC1 proteins. Two experienced pathologists who blindly examined the specimens performed immunohistochemical analysis.

### Statistical analysis

Statistical analyses were performed applying SPSS software version 20.0 (IBM Corp., NY, USA). The data were evaluated using the chi-square test, and comparisons between two groups were performed using an independent t-test, two-tailed. Significance was defined as a *p* value <0.05.

## Results

**Fig. 1:**
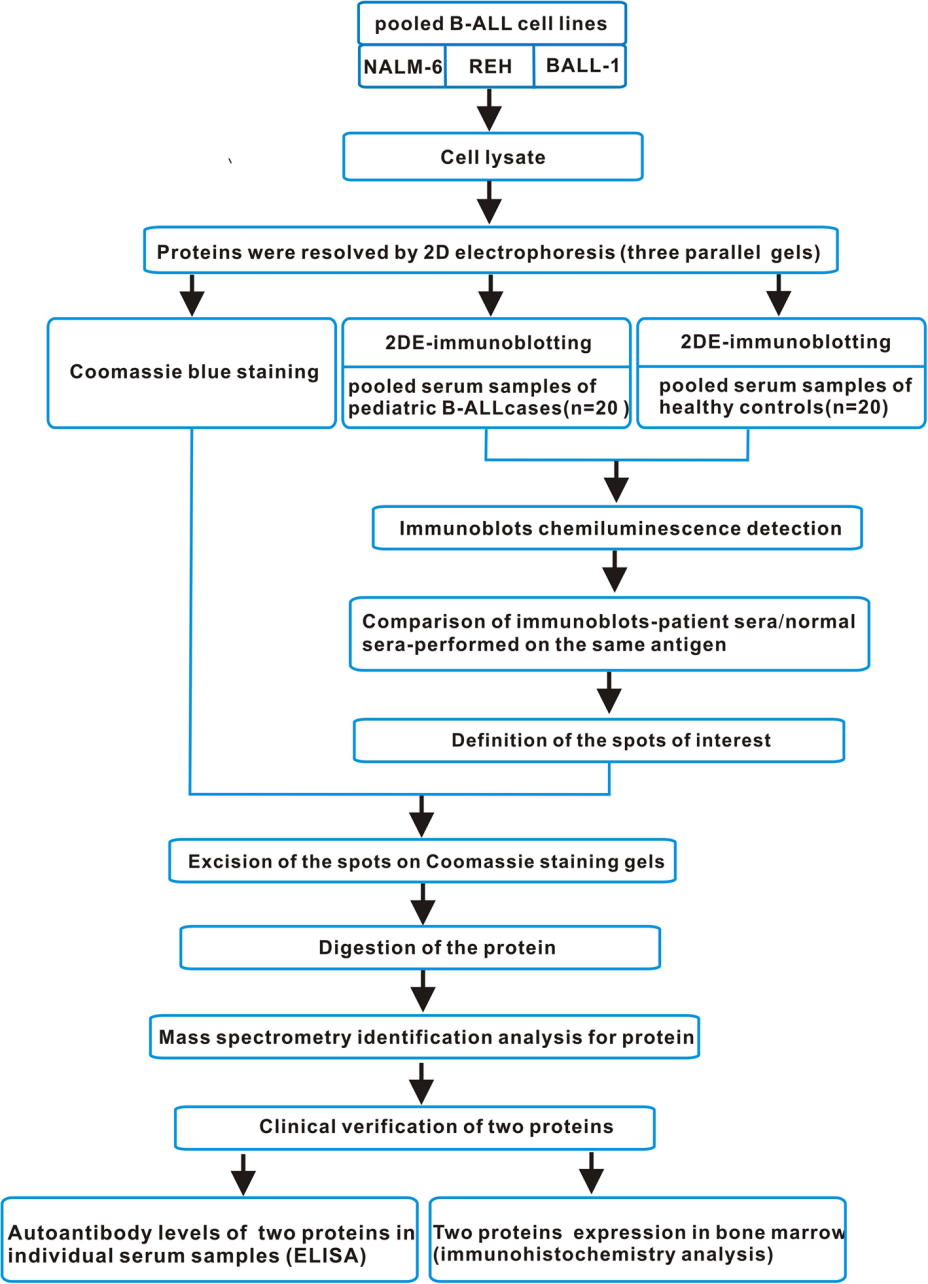
The overview of the experimental workflow. In brief, we separated total proteins extracted from the three B-ALL cell lines (NALM-6、REH and BALL-1) by 2-DE. Next, one of the three parallel 2-DE gels was visualized by Coomassie blue staining while the others were transferred onto PVDF membrances. Then, the membranes were incubated with mixed serum samples from 20 B-ALL or 20 healthy controls. After a differential analysis between the blots obtained with patients and controls sera, 6 protein spots of interest were excised from the 2-DE gels, digested by trypsin, and identified by mass spectrometry. The autoantibodies of α-enolase and voltage-dependent anion-selective channel protein 1(VDAC1) were further validated by ELISA, respectively. The candidate antigens α-enolase and VDAC1 were further validated in bone marrow by immunohistochemistry, respectively

In this study, we used SERPA approach for the detection of serum autoantibodies in B-ALL children, followed by clinical validation by ELISA and immunohistochemical detection. The whole workflow of the study was shown in Fig. 1.

### Identification of autoantibodies by SERPA

**Fig. 2:**
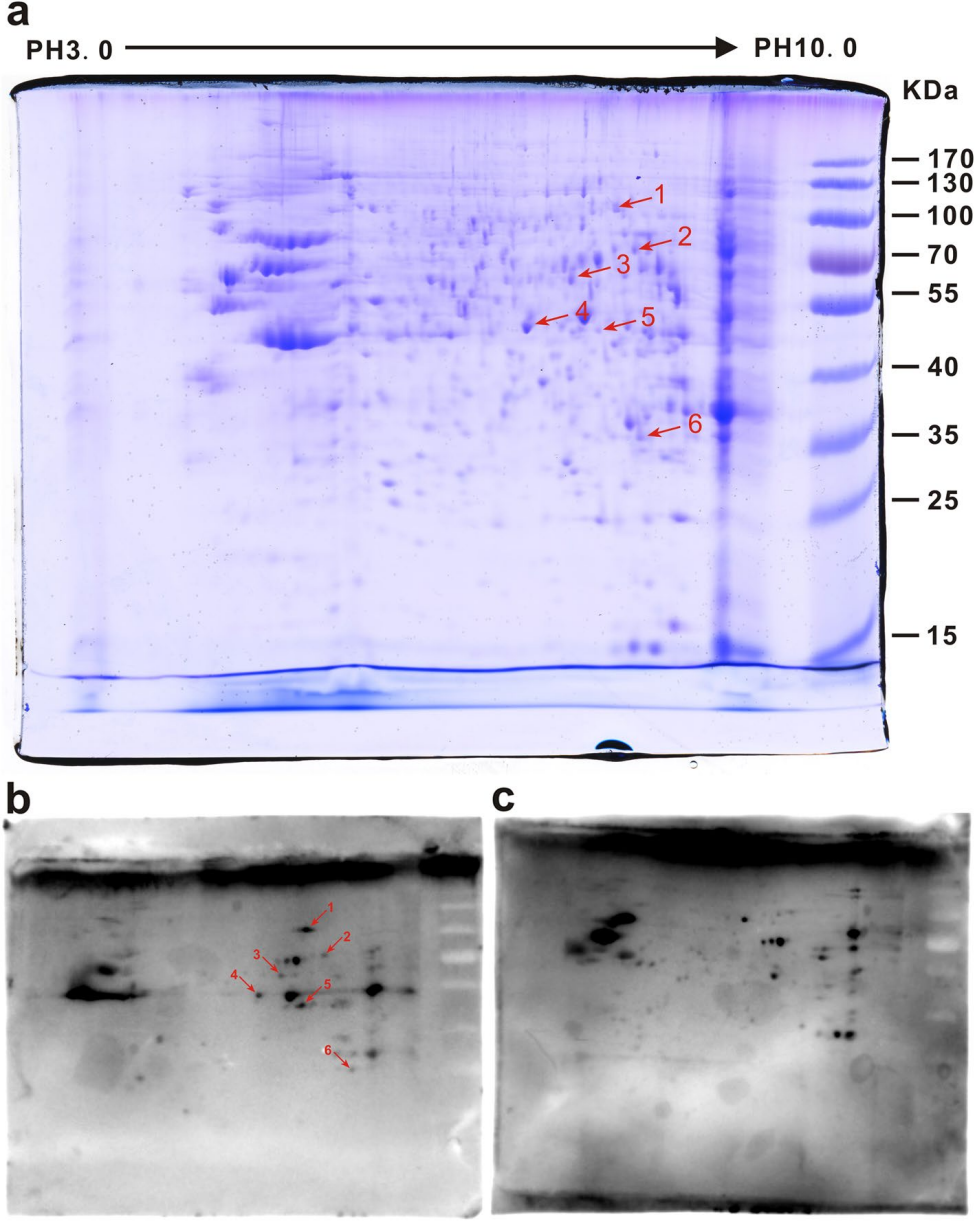
Analysis of the autoantibody response in B-ALL using 2-D immunoblotting. 2-D Immunoblotting images showed 6 different immunoreactive protein spots (marked with arrows) specifically recognized by serum from children with B-ALL. Mixed proteins from 3 B-ALL cell were separated by 2-DE and visualized by Coomassie blue staining (**a**). Mixed proteins were separated by 2-D PAGE and transferred to PVDF membranes. Then, the membranes were incubated with pooled serum from children with B-ALL (**b**). PVDF membrane incubated with pooled serum from normal controls (**c**)

**Fig. 3:**
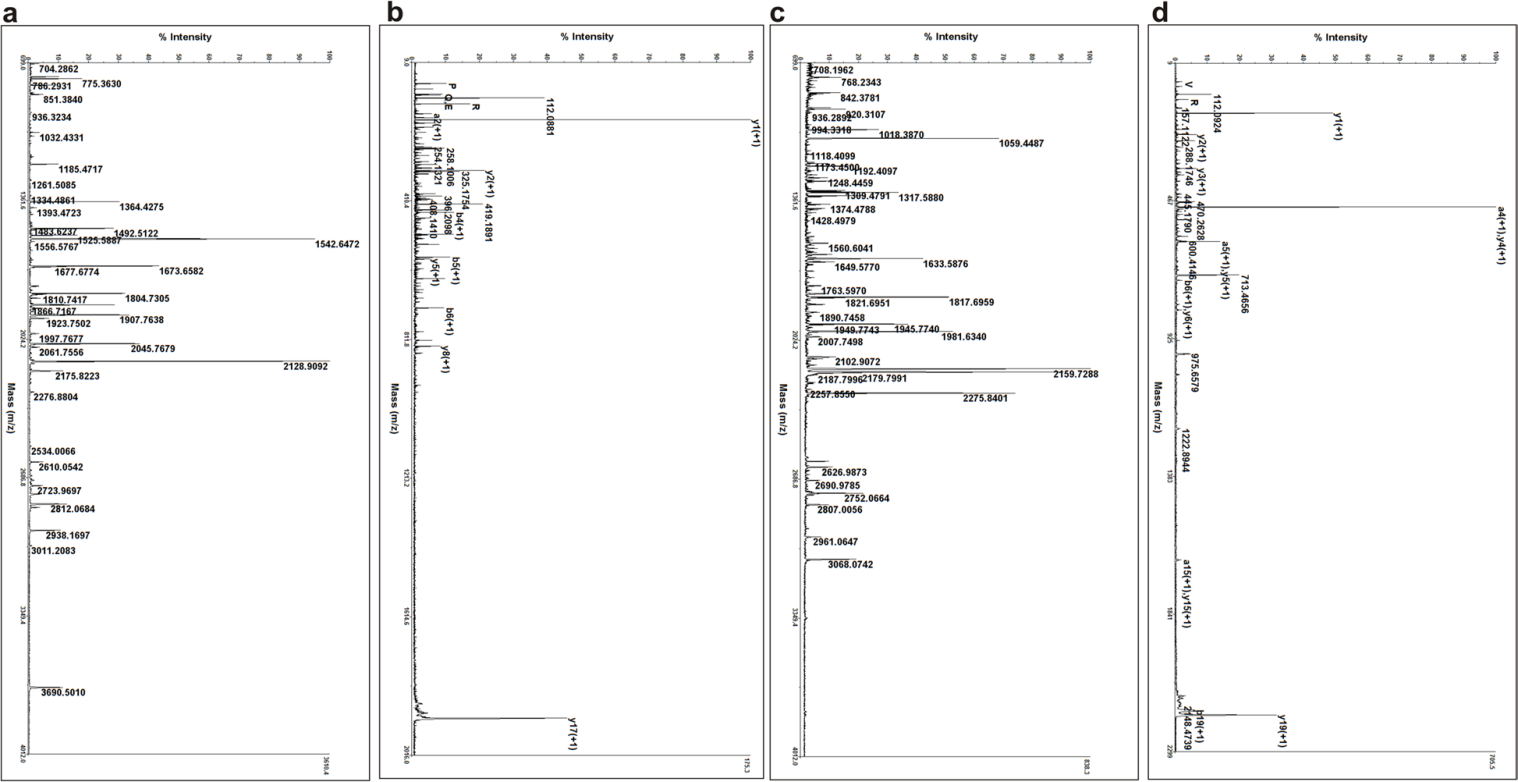
The peptide mass fingerprinting (PMF) map and MS/MS map of α-enolase and VDAC1. **a** PMF map of protein spot α-enolase. **b** MS/MS map of protein spot α-enolase. **c** PMF map of protein VDAC1. **d** MS/MS map of protein VDAC1. The x-axis represents the mass charge ratio (m/z), the Y-axis represents the relative intensity, and the number beside the peak represents the mass number of a single isotope peak [M+H]^+^ of the peptide fragment

To identify serological autoantibodies against antigens from B-ALL cells, a mixture of the total proteins from three human B-ALL cells was separated by 2-DE and transferred onto PVDF membranes or visualized by Coomassie blue staining (Fig. 2a). Subsequently the PVDF membranes were incubated with pooled serum from 20 B-ALL patients or 20 matched normal controls. Full size 2-DE gel and Western blot images were provided in Additional file (1) The antigenic protein profiles of each 2-D immunoblot were compared and matched to the original 2-DE. As a consequence, a total of 6 protein spots were significantly and specifically recognized by serum from B-ALL children, but no reactive spots were observed in the samples probed with serum from normal controls (Fig. 2b and c). These spots were marked with an arrow in the Coomassie-stained 2-D gel and 2-D immunoblots (Fig. 2a and b). Then, the proteins were subjected to MS analysis, and candidate autoantigens were identified by a database search as detailed in Table 2. The sequences of identified peptides and the raw data of MS were listed in Additional file 2 and 3, respectively. Figure 3 showed the peptide mass fingerprinting (PMF) map and MS/MS map of protein spots 4 and 6. Identification of multiple proteins from a single gel spot may be due to lower resolution of proteins through broad pH range used for iso-electric focusing in 2-DE.

**Table 2:** Summary of identified proteins from immunoreactive protein spots by MS analysis

Spot No.	Accession No.	Protein name	Theoretical pI/Mw	Mass Weight	No. of Peptides matched	Score
1	gi|20,072,188	Aconitase 2	7.62/ 85564.57	85,511	30	257
2	gi|4,323,587	AIF	9.04/66900.61	66,859	15	116
	gi|21,619,168	SYNCRIP	7.17/ 58735.75	58,699	13	94
3	gi|71,042,410	DLD	6.35/ 50147.55	50,116	15	122
	gi|109,948,304	WD40 repeat-containing protein SMU1	6.74/ 57543.89	57,507	15	162
4	gi|119,339	Alpha-enolase	7.01/ 47168.96	47,139	17	253
	gi|704,416	elongation factor Tu	7.69/ 49540.55	49,509	30	542
5	gi|7,542,837	MCAD	8.57/ 47007.78	46,978	24	274
6	gi|130,683	VDAC1	8.62/ 30772.60	30,754	11	170

### Clinical validation by ELISA using recombinant proteins

From the identified candidates, α-enolase and VDAC1 were selected for clinical verification. We screened 30 sera from children with newly diagnosed B-ALL and 25 healthy controls by ELISA for the presence of antibodies against α-enolase and VDAC1. Table 3 showed the frequency of antibodies against α-enolase and VDAC1 in sera from 30 patients with B-ALL and 25 healthy children. Of the 30 sera with B-ALL analyzed, 8 (27%) and 7 (23%) were reactive with α-enolase and VDAC1, only 1 (1.3%) and 0 was positive in healthy children sera, respectively. Statistical analysis indicated that there were significant differences (*p* < 0.05) of autoantibodies against α-enolase and VDAC1 frequency between B-ALL and healthy children. These results suggested that these proteins played roles in the carcinogenesis of B-ALL.

**Table 3:** Frequency of autoantibodies responses against α-enolase and VDAC1 in serum by ELISA

Group	n	Autoantibody positive(%)	
		α-enolase*	VDAC1*
B-ALL patients	30	8 (27%)	7(23%)
Healthy controls	25	1(4%)	0(0/25)

*p-values relative to normal controls, *p* < 0.05

### Immunohistochemical detection of α-enolase and VDAC1

**Fig. 4:**
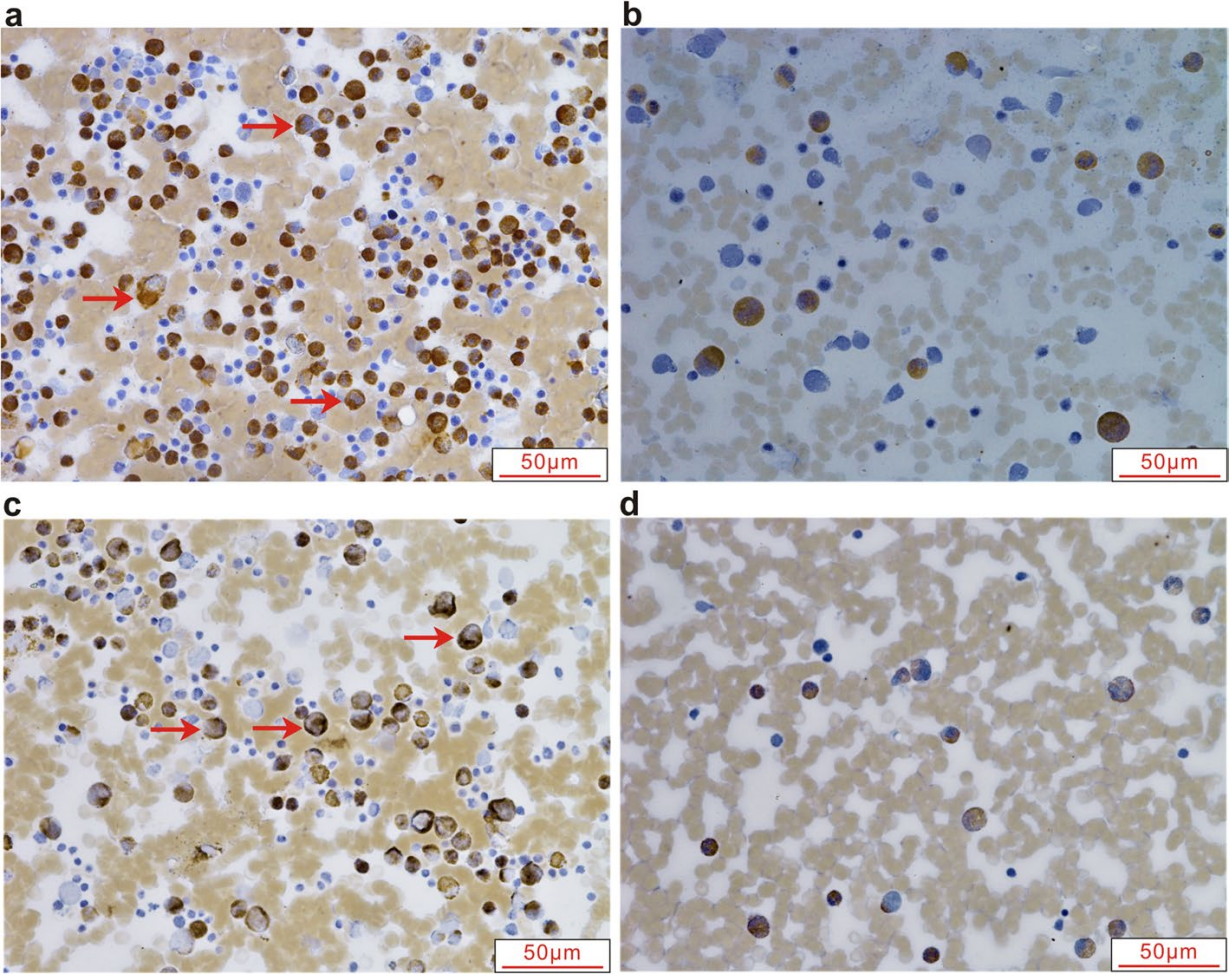
Representative immunohistochemical images showing expression of α-enolase (**a** and **b**) and VDAC1(**c** and **d**) in B-ALL and controls(400X. a and c: B-ALL group; b and d: Control group). Immunohistochemical analysis performed using BM from 20 B-ALL children showed positive expression (marked with arrows) of α-enolase and VDAC1 in 95% and 85% of B-ALL patients, respectively, whereas 10 controls presented negative

The expression of α-enolase and VDAC1 in bone marrow smears of 20 children with B-ALL and 10 controls were performed using immunohistochemistry by immunohistochemical analysis. The expression of α-enolase and VDAC1 was positive in 95% and 85% of B-ALL patients, respectively, but the control group presented negative (Fig. 4). α-enolase and VDAC1 proteins were mainly expressed in the cytoplasm of cells.

## Discussion

Growing evidence has confirmed that there was a specific immune response in tumor patients, including patients with prostate cancer, hepatocellular carcinoma, breast cancer, lung cancer, pancreatic cancer and so on [19]. Reports also demonstrated that the autoantibodies were detectable during the transition to malignancy [20, 21]. Different from other markers, autoantibodies emerged in the early process of oncogenesis, and they were shown in serum before TAAs can be detected [22]; thus the examination of tumor autoantibodies using minimally invasive methods, had huge potentiality in early diagnosis, especially for asymptomatic patients. This can facilitate early detection and treatment, thereby protecting patients from early death.

In the earliest stage of leukemia, the number of leukemic cells in the peripheral blood is very small, and the patients have almost no clinical symptoms. So it is difficult to detect leukemia-associated antigens in peripheral blood due to their low expression, but pathological changes could be detected by the immune system from the earliest stages. Immune system responds to them quickly by producing a large number of specific autoantibodies against antigens. Moreover, the prognosis of B-ALL was not only related to various factors, such as age, sex, and molecular genetic characteristics, but also closely associated with host immune function [23]. The autoantigens were not only helpful to understand the molecular mechanism, but also useful as the target for immunotherapy of B-ALL, and the corresponding autoantibodies may be serum biomarkers for early diagnosis, disease monitoring and assessment of B-ALL prognosis.

SERPA has been widely applied as a hopeful means for screening and identifying all components of immunoreactive proteins on the basis of 2-DE, immunoblotting, and MS [24]. Autoantigens were confirmed by SERPA in a variety of illnesses, containing gastric cancer [25], colorectal cancer [10], lung cancer [14], gallbladdercarcinoma [15], prostate cancer [16] and primary open angle glaucoma [18] in the past decade. In this study, mixed proteins from three B-ALL cell lines were dissolved by 2-DE, followed by Western blot analysis using mixed serum samples from children with B-ALL and healthy controls. As a result, 6 protein spots that were significantly and specifically recognized by serum from B-ALL patients were identified. MS analysis of 6 protein spots that showed specific immunoreactivity in pediatric B-ALL led to the identification of 9 proteins. A literature survey of the identified proteins and corresponding autoantibodies was done for their expression in B-ALL or other tumors, based on which we selected α-enolase and VDAC1 for validations using individual serum samples [26–32].

We then verified the production of autoantibodies against α-enolase and VDAC1 in 30 children with B-ALL and 25 normal controls by ELISA. In the validation stage, we proved that α-enolase and VDAC1 autoantibodies were new representative targets for distinguishing patients with B-ALL from normal controls. To the best of our knowledge, this study was the first report of the existence of autoantibodies against α-enolase and VDAC1 in serum from children with B-ALL by a proteomic approach, nevertheless, the α-enolase autoantibody was not all specific to B-ALL serum and were found in the serum of patients with other tumors, as well as α-enolase antibodies were found in some autoimmune diseases [33–35]. The mechanism which α-enolase and VDAC1 induce immune response in B-ALL was unclear.

One of two proteins, α-enolase, found on the surface of cells, was is involved in several key biological processes of cancer, including proliferation, migration and invasion [36, 37]. According to previous reports, α-enolase was overexpressed in multiple tumors, including lung cancer [38], hepatocellular carcinoma [39], pancreatic cancer [40], and gastric cancer patients [41], and was proposed as a biomarker for early detection or prognosis [42]. Several reports mentioned α-enolase as a tumor-associated antigen that could induce autoantibody production in malignant tumors [28, 29]. Autoantibodies against α-enolase were also reported in acute leukemia [43], chronic myeloid leukemia [44], non-Hodgkin’s lymphoma [45] and other solid tumors [42]. A recent study demonstrated that α-enolase and Annexin A1 autoantibodies could enhance diagnostic performance in lung cancers by combining CEA and CA125 [29]. Another study suggested that α-enolase could accelerate metastasis of lung cancer cell through HGFR and WNT signaling pathway, and presented a new antibody targeting α-enolase in lung cancer [37]. In multiple myeloma, α-enolase expression negatively correlated with overall survival, and targeting α-enolase could enhance immunity and improve outcome [46]. Cui et al. found that positivity of α-enolase autoantibodies was observed in serum from 18 to 21 (86%) patients with acute leukemia (AL) and 20 of 22 (90%) healthy controls [43]. However, we found that α-enolase autoantibodies were detected in serum from 8 to 30 (27%) children with ALL and 1 of 25 (4%) healthy controls. The reasons for the differences described above may be related to subjects in the different age and leukemia groups.

Similarly, we also selected another protein, VDAC1, for verification. We demonstrated that positivity for autoantibodies against VDAC1 was observed in sera from 7 to 30 (23%) children with ALL, but no such activity was observed in 25 (0%) normal children. VDAC1 is a mitochondrial protein controlling cell growth, energy production and Ca^2+^ homeostasis [47, 48]. Moreover, VDAC1 also regulated apoptosis by mediating the release of apoptotic proteins from mitochondria and interacting with antiapoptotic proteins [47, 49]. The observation that VDAC1 was overexpressed in a variety of tumors showed that VDAC1 may be essential for cancer cell survival [47, 50, 51]. Accumulating studies have proved that abrogation of VDAC1 expression significantly inhibited tumor growth in cancers [48, 50–54], suggesting that VDAC1 may be a novel therapeutic target [55–58]. Furthermore, targeted drugs acting on VDAC1 against tumor growth and proliferation was a promising strategy for the treatment of cancer [59]. However, as far as I know, VDAC1 has never previously been reported as an autoantigen and may become a new target antigen. Antibodies against VDAC1 can also be found in the serum of B-ALL children, indicating that VDAC1 triggered autoimmunity, and leaded to elevation of VDAC1 autoantibody. However, the exact mechanisms required further study. Our results proved that VDAC1 may be a promising antigen which could be immunogenic, and autoantibodies against VDAC1 might serve as a potential biomarker for B-ALL.

## Conclusions

In summary, the study applied SERPA method for the screening of serum autoantibodies in children with B-ALL. Our data on clinical verification suggested that α-enolase and VDAC1 autoantibodies were promising biomarkers for children with B-ALL, and measuring serum autoantibodies against α-enolase and VDAC1 may show promise for clinical application in terms of diagnosis, immunological surveillance, treatment and prognosis of children with B-ALL. However, the sample size of B-ALL children used in this study was small and the analysis may be carried out further in a larger cohort of samples.

## Supplementary Information

**Additional file 1.** Two-Dimensional electrophoresis (2-DE) gel and Western blot images.

**Additional file 2.** The sequences of identified peptides.

**Additional file 3.** Raw data of mass spectrometry.

## Data Availability

Raw data file (Additional file 3) can be accessed via the Data Explorer V4.5. All data supporting the conclusions of this study were included in this published article and its additional files.

## Abbreviations

2-DE: two-dimensional electrophoresis
AIF: apoptosis-inducing factor
α-enolase: alpha-enolase
B-ALL: B-cell acute lymphoblastic leukemia
BM: bone marrow
DLD: dihydrolipoamide dehydrogenase
ELISA: enzyme-linked immunosorbent assay
MS: mass spectrometry
MCAD: medium-chain acyl-CoA dehydrogenase
PBS: phosphate-buffered saline
PMF: peptide mass fingerprinting
PVDF: polyvinylidene fluoride
SERPA: serological proteome analysis
SYNCRIP: synaptotagmin binding cytoplasmic RNA interacting protein
TAAs: stumor-associated antigens
VDAC1: voltage-dependent anion-selective channel protein 1
