# Supplementary material for: Identification and validation of serum autoantibodies in children with B-cell acute lymphoblastic leukemia by serological proteome analysis
Source: Proteome Sci. 2022 Feb 2;20:3. doi: 10.1186/s12953-021-00184-w (PMC8808998; doi:10.1186/s12953-021-00184-w)

**Additional file 2 – The sequences of identified peptides.**

# **Spot 1**

# **Aconitase 2, mitochondrial [Homo sapiens]**

ORIGIN

1 mapysllvtr lqkalgvrqy hvasvlcqra kvamshfepn eyihydllek ninivrkrln

61 rpltlsekiv yghlddpasq eiergksylr lrpdrvamqd ataqmamlqf issglskvav

121 pstihcdhli eaqvggekdl rrakdinqev ynflatagak ygvgfwkpgs giihqiilen

181 yaypgvllig tdshtpnggg lggicirvgg adavdvmagi pwelkcpkvi gvkltgslsg

241 wsspkdvilk vagiltvkgg tgaiveyhgh gvdsisctgm aticnmgaei gattsvfpyn

301 hrmkkylskt gredianlad efkdhlvpdp gchydqliei nlselkphin gpftpdlahp

361 vaevgkvaek egwpldirvg ligsctnssy edmgrsaava kqalahglkc ksqftitpgs

421 eqiratierd gyaqilrdlg givlanacgp cigqwdrkdi kkgekntivt synrnftgrn

481 danpethafv tspeivtala iagtlkfnpe tdyltgtdgk kfrleapdad elpkgefdpg

541 qdtyqhppkd ssgqhvdvsp tsqrlqllep fdkwdgkdle dlqilikvkg kcttdhisaa

601 gpwlkfrghl dnisnnllig ainiengkan svrnavtqef gpvpdtaryy kkhgirwvvi

661 gdenygegss rehaaleprh lggraiitks farihetnlk kqgllpltfa dpadynkihp

721 vdkltiqglk dftpgkplkc iikhpngtqe tillnhtfne tqiewfrags alnrmkelqq


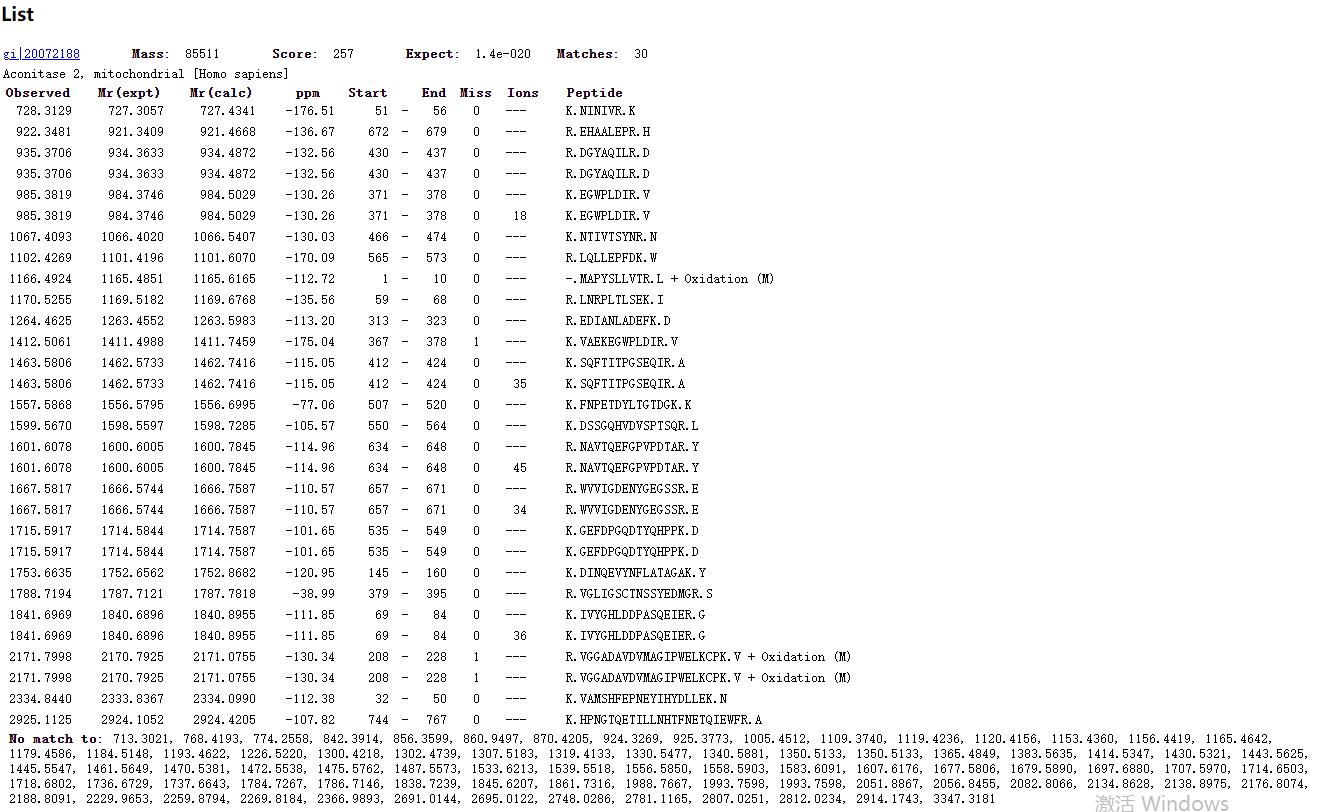


# **Spot 2**

# **apoptosis-inducing factor AIF [Homo sapiens]**

ORIGIN

1 mfrcgglaag alkqklvplv rtvcvrsprq rnrlpgnlfq rwhvplelqm trqmassgas

61 ggkidnsvlv livglstvga gayayktmke dekryneris glgltpeqkq kkaalsaseg

121 eevpqdkaps hvpflliggg taafaaarsi rardpgarvl ivsedpelpy mrpplskelw

181 fsddpnvtkt lrfkqwngke rsiyfqppsf yvsaqdlphi enggvavltg kkvvqldvrd

241 nmvklndgsq ityekcliat ggtprslsai dragaevksr ttlfrkigdf rslekisrev

301 ksitiigggf lgselacalg rkaralgtev iqlfpekgnm gkilpeylsn wtmekvrreg

361 vkvmpnaivq svgvssgkll iklkdgrkve tdhivaavgl epnvelaktg gleidsdfgg

421 frvnaelqar sniwvagdaa cfydiklgrr rvehhdhavv sgrlagenmt gaakpywhqs

481 mfwsdlgpdv gyeaiglvds slptvgvfak ataqdnpksa teqsgtgirs esetesease

541 itippstpav pqapvqgedy gkgvifylrd kvvvgivlwn ifnrmpiark iikdgeqhed

601 lnevaklfni hed


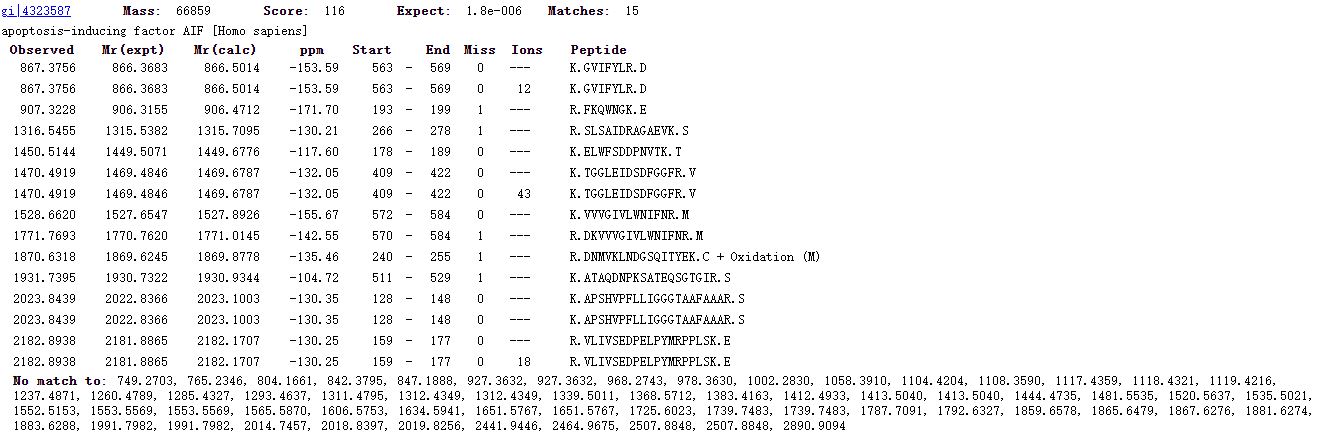


# **SYNCRIP protein [Homo sapiens]**

# ORIGIN

# 1 matehvngng teepmdttsa vihsenfqtl ldaglpqkva ekldeiyvag lvahsdlder

# 61 aiealkefne dgalavlqqf kdsdlshvqn ksaflcgvmk tyrqrekqgt kvadsskgpd

# 121 eakikaller tgytldvttg qrkyggpppd svysgqqpsv gteifvgkip rdlfedelvp

# 181 lfekagpiwd lrlmmdpltg lnrgyafvtf ctkeaaqeav klynnheirs gkhigvcisv

# 241 annrlfvgsi pksktkeqil eefskvtegl tdvilyhqpd dkkknrgfcf leyedhktaa

# 301 qvkvlfvrnl antvteeile kafsqfgkle rvkklkdyaf ihfderdgav kameemngkd

# 361 legenieivf akppdqkrke rkaqrqaakn qmyddyyyyg pphmppptrg rgrggrggyg

# 421 yppdyygyed yydyygydyh nyrggyedpy ygyedfqvga rgrggrgarg aapsrgrgaa

# 481 pprgragysq rggpgsargv rgarggaqqq rgrgqgkgve agpdllq

#
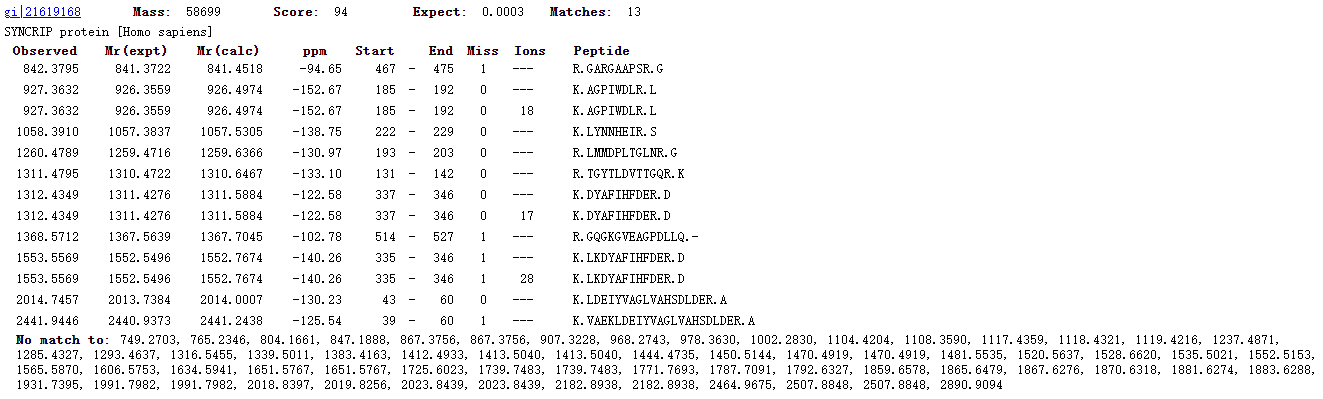


# **Spot 3**

# **Chain H, Dihydrolipoyl dehydrogenase**

ORIGIN

1 adqpidadvt vigsgpggyv aaikaaqlgf ktvcieknet lggtclnvgc ipskallnns

61 hyyhmahgtd fasrgiemse vrlnldkmme qkstavkalt ggiahlfkqn kvvhvngygk

121 itgknqvtat kadggtqvid tkniliatgs evtpfpgiti dedtivsstg alslkkvpek

181 mvvigagvig velgsvwqrl gadvtavefl ghvggvgidm eisknfqril qkqgfkfkln

241 tkvtgatkks dgkidvsiea asggkaevit cdvllvcigr rpftknlgle elgieldprg

301 ripvntrfqt kipniyaigd vvagpmlahk aedegiicve gmaggavhid yncvpsviyt

361 hpevawvgks eeqlkeegie ykvgkfpfaa nsraktnadt dgmvkilgqk stdrvlgahi

421 lgpgagemvn eaalaleyga scediarvch ahptlseafr eanlaasfgk sinf

#
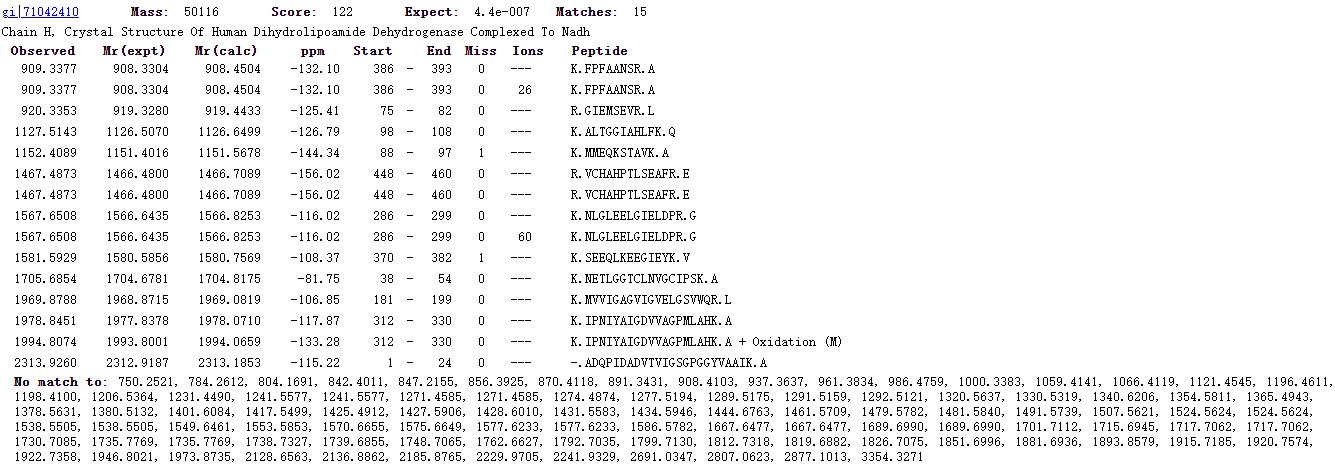


# **WD40 repeat-containing protein SMU1 [Homo sapiens]**

# ORIGIN

# 1 msieiessdv irlimqylke nslhralatl qeettvslnt vdsiesfvad insghwdtvl

# 61 qaiqslklpd ktlidlyeqv vlelielrel gaarsllrqt dpmimlkqtq peryihlenl

# 121 larsyfdpre aypdgsskek rraaiaqala gevsvvppsr lmallgqalk wqqhqgllpp

# 181 gmtidlfrgk aavkdveeek fptqlsrhik fgqkshveca rfspdgqylv tgsvdgfiev

# 241 wnfttgkirk dlkyqaqdnf mmmddavlcm cfsrdtemla tgaqdgkikv wkiqsgqclr

# 301 rferahskgv tclsfskdss qilsasfdqt irihglksgk tlkefrghss fvneatftqd

# 361 ghyiisassd gtvkiwnmkt tecsntfksl gstagtditv nsvillpknp ehfvvcnrsn

# 421 tvvimnmqgq ivrsfssgkr eggdfvccal sprgewiycv gedfvlycfs tvtgklertl

# 481 tvhekdvigi ahhphqnlia tysedgllkl wkp


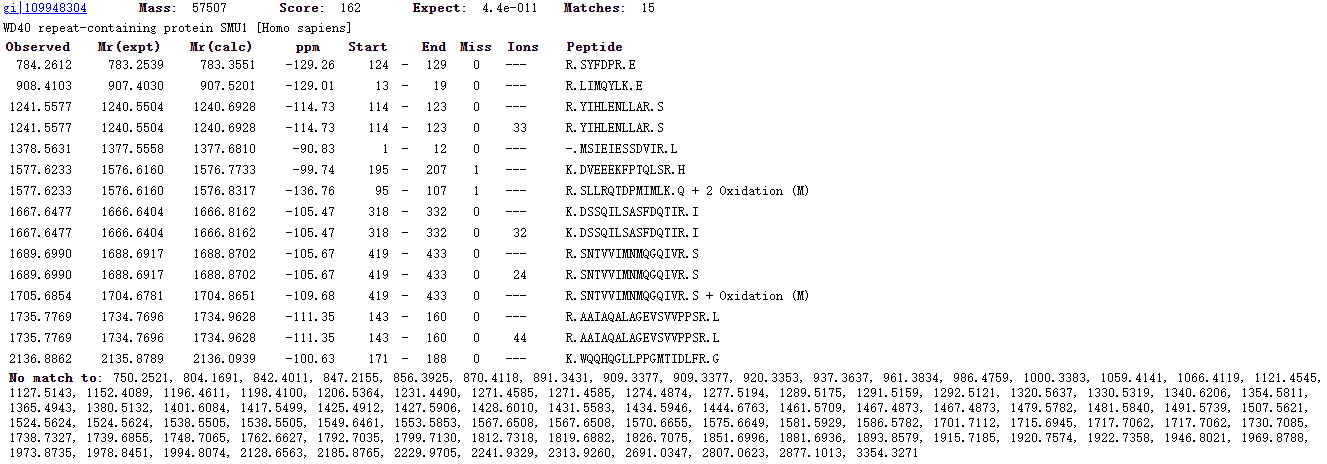


# **Spot 4**

# **Alpha-enolase**

ORIGIN

1 msilkihare ifdsrgnptv evdlftskgl fraavpsgas tgiyealelr dndktrymgk

61 gvskavehin ktiapalvsk klnvteqeki dklmiemdgt enkskfgana ilgvslavck

121 agavekgvpl yrhiadlagn sevilpvpaf nvinggshag nklamqefmi lpvgaanfre

181 amrigaevyh nlknvikeky gkdatnvgde ggfapnilen keglellkta igkagytdkv

241 vigmdvaase ffrsgkydld fkspddpsry ispdqladly ksfikdypvv siedpfdqdd

301 wgawqkftas agiqvvgddl tvtnpkriak avnekscncl llkvnqigsv teslqackla

361 qangwgvmvs hrsgetedtf iadlvvglct gqiktgapcr serlakynql lrieeelgsk

421 akfagrnfrn plak


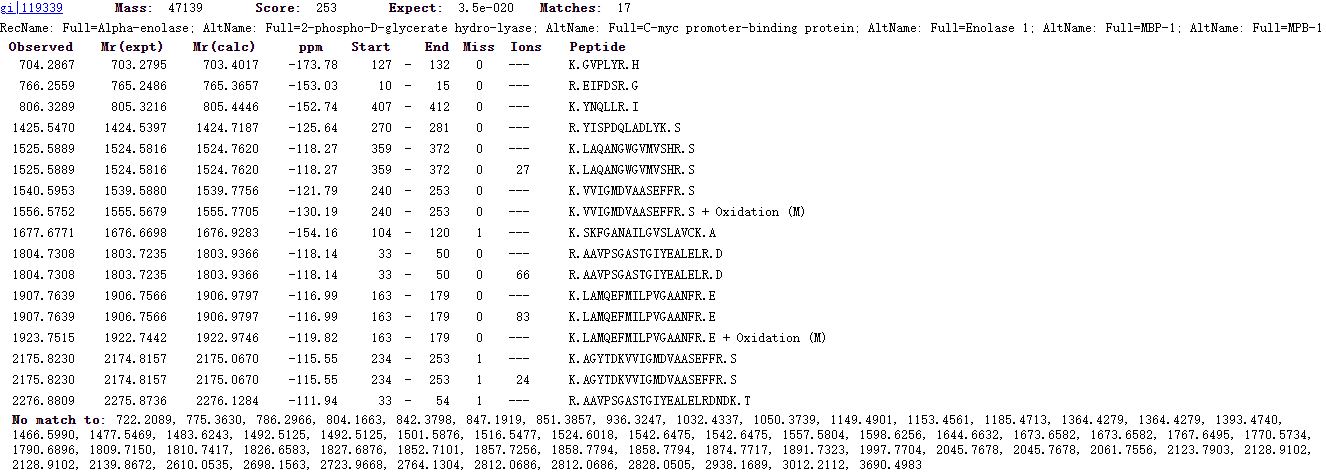


# **elongation factor Tu [Homo sapiens]**

ORIGIN

1 maaatllrat phfsglaagr tfllqgllrl lkapalpllc rglaveakkt yvrdkphvnv

61 gtighvdhgk ttltaaitki laegggakfk kyeeidnape erargitina ahveystaar

121 hyahtdcpgh adyvknmitg tapldgcilv vaandgpmpq trehlllarq igvehvvvyv

181 nkadavqdse mvelveleir elltefgykg eetpvivgsa lcalegrdpe lglksvqkll

241 davdtyipvp ardlekpfll pveavysvpg rgtvvtgtle rgilkkgdec ellghsknir

301 tvvtgiemfh ksleraeagd nlgalvrglk redlrrglvm vkpgsikphq kveaqvyils

361 keeggrhkpf vshfmpvmfs ltwnmacrii lppekelamp gedlkfnlil rqpmilekgq

421 rftlrdgnrt igtglvtntl amteeeknik wg


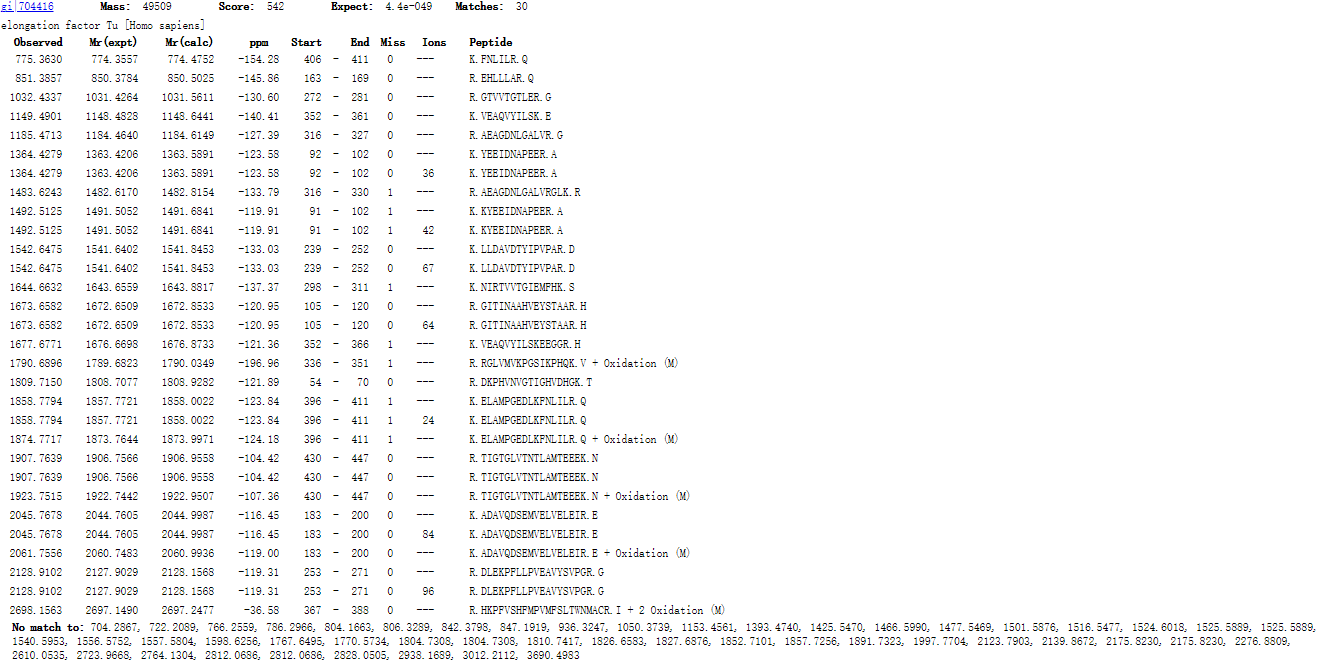


# **Spot 5**

# **medium-chain acyl-CoA dehydrogenase [Homo sapiens]**

ORIGIN

1 maagfgrccr cslqvlrsis rfhwrsqhtk anrqrepglg fsfefteqqk efqatarkfa

61 reeiipvaae ydktgeypvp lirrawelgl mnthipencg glglgtfdac liseelaygc

121 tgvqtaiegn slgqmpiiia gndqqkkkyl grmteeplmc aycvtepgag sdvagiktka

181 ekkgdeyiin gqkmwitngg kanwyfllar sdpdpkapan kaftgfivea dtpgiqigrk

241 elnmgqrcsd trgivfedvk vpkenvligd gagfkvamga fdktrpvvaa gavglaqral

301 deatkyaler ktfgkllveh qaisfmlaem amkvelarms yqraawevds grrntyyast

361 akafagdian qlatdavqil ggngfnteyp veklmrdaki yqiyegtsqi qrlivarehi

421 dkykn


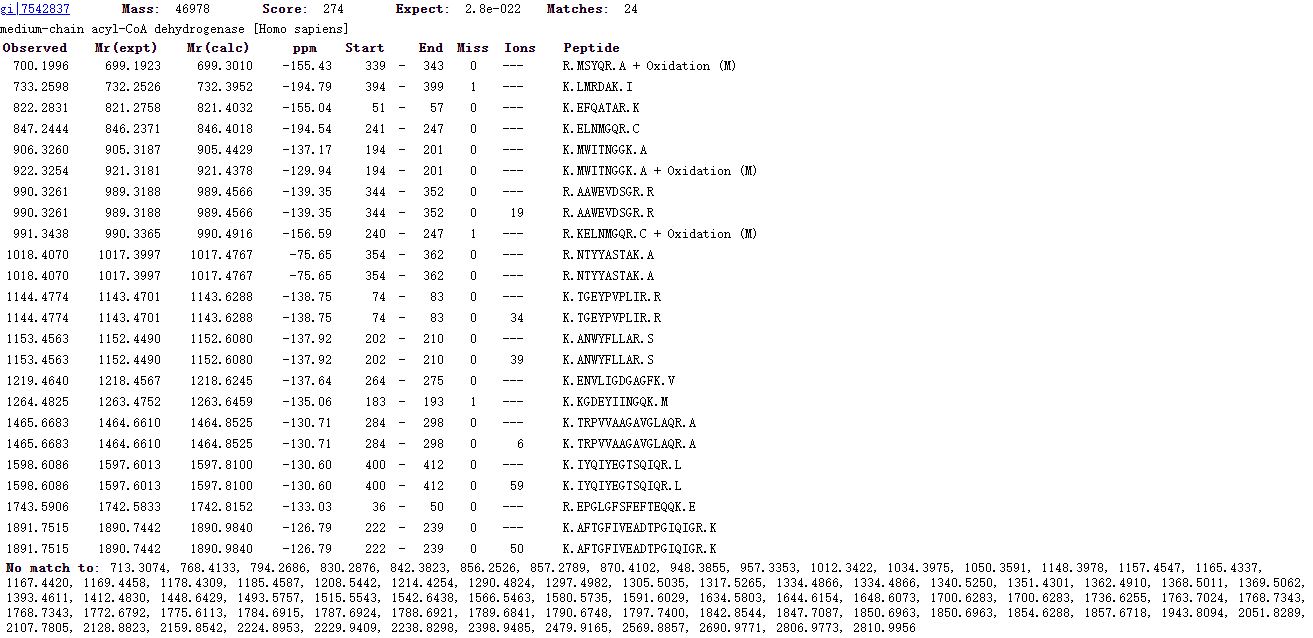


# **Spot 6**

# **Voltage-dependent anion-selective channel protein 1**

ORIGIN

1 mavpptyadl gksardvftk gygfglikld lktksengle ftssgsante ttkvtgslet

61 kyrwteyglt ftekwntdnt lgteitvedq larglkltfd ssfspntgkk nakiktgykr

121 ehinlgcdmd fdiagpsirg alvlgyegwl agyqmnfeta ksrvtqsnfa vgyktdefql

181 htnvndgtef ggsiyqkvnk kletavnlaw tagnsntrfg iaakyqidpd acfsakvnns

241 sliglgytqt lkpgikltls alldgknvna gghklglgle fqa


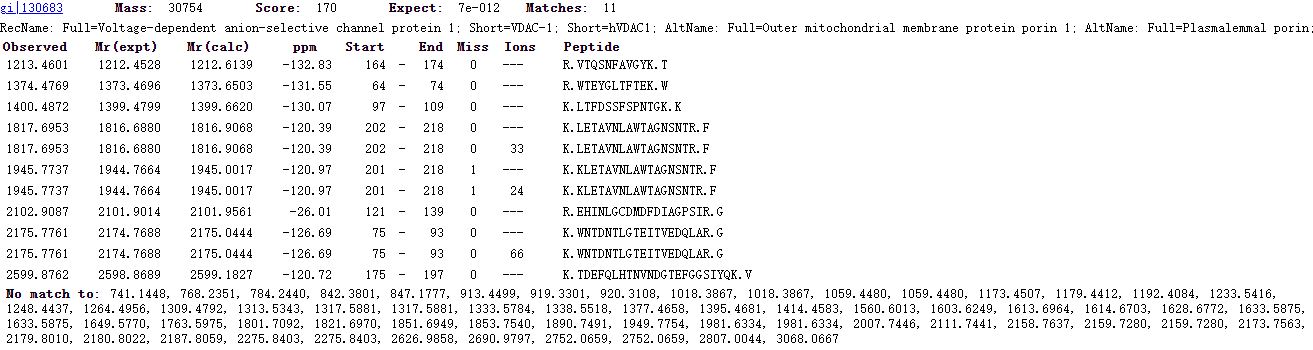

Supplement: Supplementary file 2 — Additional file 2. The sequences of identified peptides. [file 12953_2021_184_MOESM2_ESM.doc]
