# Supplementary material for: Identification and validation of serum autoantibodies in children with B-cell acute lymphoblastic leukemia by serological proteome analysis
Source: Proteome Sci. 2022 Feb 2;20:3. doi: 10.1186/s12953-021-00184-w (PMC8808998; doi:10.1186/s12953-021-00184-w)
Supplement: Supplementary file 1 — Additional file 1. Two-Dimensional electrophoresis (2-DE) gel and Western blot images. [file 12953_2021_184_MOESM1_ESM.doc]

**Additional file 1 – Two-Dimensional electrophoresis (2-DE) gel and Western blot images**

**Gels # 1-10: 2-DE gel**

Gel # 1

Gel # 2

Gel # 3

Gel # 4

Gel # 5

Gel # 6

Gel # 7

Gel # 8

Gel # 9

Gel # 10

**Gels # 11 - 16: 2-DE Western blot images**

**Gels # 11-13: PVDF membrane incubated with pooled serum from children with B-ALL**

Gels # 11

Gels # 12

Gels # 13

**Gels # 14 - 16:PVDF membrane incubated with pooled serum from children with pooled serum from normal controls.**

Gels # 14

Gels # 15

Gels # 16
